# Improving Outcomes by Implementing a Pressure Ulcer Prevention Program (PUPP): Going beyond the Basics

**DOI:** 10.3390/healthcare3030574

**Published:** 2015-07-17

**Authors:** Amparo Cano, Debbie Anglade, Hope Stamp, Fortunata Joaquin, Jennifer A. Lopez, Lori Lupe, Steven P. Schmidt, Daniel L. Young

**Affiliations:** 1 University of Miami Hospital, Miami FL, 1400 NW 12th Avenue, Miami, FL 33136, USA; E-Mails: HStamp@med.miami.edu (H.S.); FJoaquin@med.miami.edu (F.J.); JLopez13@med.miami.edu (J.A.L.); LLupe@med.miami.edu (L.L.); 2 School of Nursing and Health Studies, University of Miami, Brunson Drive, Coral Gables, FL 33146, USA; E-Mail: d.anglade@miami.edu; 3 Department of Pharmaceutical Sciences, Northeast Ohio Medical University, Rootstown, OH 44272, USA; E-Mail: sschmidt@neomed.edu; 4 Department of Physical Therapy, University of Nevada Las Vegas, 4505 Maryland Pkwy., Box 453029, Las Vegas, NV 89154, USA; E-Mail: daniel.young@unlv.edu

**Keywords:** pressure ulcer prevention, hospital-acquired pressure ulcers, pressure ulcer prevention education, skin care

## Abstract

A multidisciplinary process improvement program was initiated at the University of Miami Hospital (UMH) in 2009 to identify the prevalence of hospital-acquired pressure ulcers (HAPU) at the institution and to implement interventions to reduce the incidence of HAPU. This deliberate and thoughtful committee-driven process evaluated care, monitored results, and designed evidence-based strategic initiatives to manage and reduce the rate of HAPU. As a result all inpatient beds were replaced with support surfaces, updated care delivery protocols were created, and monitored, turning schedules were addressed, and a wound, ostomy, and continence (WOC) nurse and support staff were hired. These initial interventions resulted in a decrease in the prevalence of HAPU at UMH from 11.7% of stage II to IV ulcers in the second quarter, 2009 to 2.1% the third quarter. The rate remained at or near the 2009 UMH benchmark of 3.1% until the first quarter of 2012 when the rate rose to 4.1%. At that time new skin products were introduced into practice and continuing re-education was provided. The rate of HAPU dropped to 2.76% by the second quarter of 2012 and has remained steadily low at 1%–2% for nine consecutive quarters.

## 1. Introduction

In the United States, the occurrence of pressure ulcers (PU) annually affects as many as 2.5 million patients and the development of these pressure ulcers may lead to devastating consequences for patients that significantly reduce quality of life and which may increase mortality [1,2]. Hospital admissions due to PU are 75% higher than admissions for any other medical conditions and the consequences of PU development in hospitalized patients are particularly dire. Patients with HAPU experience increased lengths of hospital stays by 7–10 days and are three times more likely to be discharged to long-term care facilities, and mortality of these patients is twice that of patients without HAPU [3,4,5]. HAPU is a common medical complication in the acute care setting with rates between 5% and 6% [6]. According to the 2014 International Hill-Rom Prevalence Survey, a national and international survey of 104,485 patients, the overall HAPU rate was reported at 3.8%. However, higher rates of HAPU were found in patients in Spinal Cord Units (10.7%), Critical Care Units (8.6%), Burn Units (7.3%), and Respiratory/Pulmonary units (7.2%) [6]. Throughout recent years, HAPU rates have remained between 4.8% (2010) and 3.7% (2012) and with only slight fluctuations [7].

The economic costs of treatment for PUs are significant. The cost of care for one PU is between $500 and $70,000, depending on the stage [8,9]. The American Journal of Surgery calculated that the cost of treatment for a stage IV HAPU totals $129 K and the cost of a present on admission (POA) PU is $124 K over an average of four admissions [10]. Several studies on PU cost concludes that cost of prevention is less expensive than cost of treatment of a pressure ulcer [11,12,13]. Of all US dollars spent in healthcare, PU accounts for almost 2% of the total cost with Medicare being the largest payer for all adult hospitalizations due to PU [14,15]. The cost of PU care is likely to increase exponentially as life expectancy increases in the future [9].

In 2010, the Centers for Medicare & Medicaid Services (CMS), urged by the 2010 Patient Protection and Affordable Care Act (ACA), imposed upon healthcare facilities new and strenuous policies to control costs and heighten quality of care. One such program is related to payment provisions for preventable hospital-acquired conditions such as HAPU. HAPU are considered preventable events and Medicare applies payment penalties to the bottom 25% lowest performing hospitals—a measure that was implemented in 2015 [16,17]. However, based on a consensus statement by the Wound Ostomy and Continence Nurses Society “unavoidable pressure ulcers do occur” [18] (p. 328).

Unavoidable Pressure ulcers as defined by Black *et al.* are “those pressure ulcers which develop in patients hemodynamically unstable, terminally ill, have certain medical devices, and are non-adherent with artificial nutrition or repositioning” [19].

The National Pressure Ulcer Advisory Panel and European Pressure Ulcer Advisory panel (NPUAP/EPUAP) defines a pressure ulcer (PU) as: “A localized injury to the skin and/or underlying tissue usually over a bony prominence, as a result of pressure, or pressure in combination with shear. A number of contributing or confounding factors are also associated with pressure ulcers; the significance of these factors has yet to be elucidated” [20].

The most consistent risk factors implicated in the development of PU are: Altered mobility and altered sensory perception, skin exposure to moisture, decrease in physical activity, inadequate nutrition and friction and shear, factors measured by the Braden Scale [21].

However, there have been more than a hundred risk factors linked to pressure ulcer development [22]. The effects of pressure onto the skin surfaces can cause occlusion or decrease in blood vessel lumen resulting in tissue ischemia, which then develops into a PU [23]. Emerging research in tissue ischemia describes the triad of pressure, shear, and temperature working in combination as precursors of PU [24]. Additionally, in a literature review by Cox [25] vasopressors, such as norepinephrine, were put forward as a potential factor for PU development in the critical care patient population.

Nevertheless, healthcare consumers and the public at large, view the occurrence of HAPU as a lower standard of care, specifically as suboptimal nursing care. Nursing is indeed the discipline that can make the strongest impact in PU care and HAPU prevention; low rates of HAPU are associated with high quality nursing care [26]. The literature shows nurse led Performance Improvement Projects, focused on implementation of evidence-based practice preventive programs, reported significant decrease of HAPU rates [27]. McInerney, in a similar size hospital system study, reduced their total HAPU rate by 81% with a 90% reduction in heel HAPU rates with the use of a comprehensive pressure ulcer prevention program (PUPP) [28]. A study by Morehead & Blain, conducted in Intensive Care Units, reported HAPU rates were reduced to zero and that this rate was maintained for over a period of months [29].

Pressure Ulcers in hospitalized patients are a high cost, high impact medical condition that is considered to be reasonably preventable through implementation of evidence-based prevention measures. Multidisciplinary programs for prevention of pressure ulcers are effective and an emphasis on the prevention of HAPU has become the central focus in many healthcare facilities due to increased litigation, government penalties, and impact in reportable hospital performance metrics [30].

Achieving zero percent HAPU is a very difficult goal to achieve even with implementation of best practice preventive measures [31]. However, significant reduction of HAPU is attainable with a comprehensive, sustainable, and robust PUPP. Key elements of such programs include: (1) Implementation of Evidence-Based practices; (2) Evidence-Based product selection; and (3) Clinician education [32,33,34]. The main objective of the UMH project was to evaluate HAPU prevalence rates post-implementation of a robust PUPP with the motivation to reduce HAPU in the facility.

## 2. Methods

The University of Miami Hospital is a facility participant in the National Database of Nursing Quality Indicators (NDNQI) program. A retrospective review of HAPU NDNQI prevalence data, collected over a two year period, was conducted to evaluate the impact on HAPU prevalence rate post implementation of a robust PUPP.

### 2.1. The UMH PUPP Experience

The University of Miami Hospital (UMH) is a 560 bed Acute Care Academic Medical Center located in the Health District of Miami, FL. The hospital is a tertiary and quaternary referral medical center affiliated with the Miller School of Medicine. In 2009, the hospital noted a spike in the prevalence of hospital acquired pressure ulcers and developed a multidisciplinary team to evaluate the root cause of the increase and develop strategies for improvement. This 14 member team, the HAPU committee, included patient care staff from all acute care inpatient units who leads and monitors wound care in their respective areas. The HAPU committee also included nursing directors, executive directors, charge nurses, a nutritionist, and an educator. The UMH HAPU committee retained oversight of the pressure ulcer program and facilitated all pressure ulcer initiatives related to evidence-based practice in the management and prevention of HAPU at UMH. Additionally, a UMH wound care champion committee was created; which was made up of volunteer staff nurses and patient care assistants. The staff nurse wound care champions’ responsibility included, among other things, collection of pressure ulcer prevalence data. The wound care champion committee was given decision making power regarding facility wide pressure ulcer management.

### 2.2. Pressure Ulcer Prevalence

Nurses at UMH utilize the evidence based Braden scale to evaluate patients’ pressure ulcer risk. The Braden Scale consists of six sub scales: sensory perception, moisture, activity, mobility, nutrition, and friction/shear. The total score can range from 6 to 23; a lower score indicates a higher risk. A score of 18 or below flags patients as at risk for pressure ulcers. In addition, nurses are encouraged to look at the scores in each subscale and to assess the patient as a whole. Braden Score Sub scales are: (Low Risk 15–18), (Moderate Risk 13–14), (High risk 10–12) and (Very High Risk 6–9) Interventions are provided based on sub scores and patient medical condition.

Pressure ulcer prevalence surveys at UMH are conducted monthly, on every third Wednesday of each month, by the wound care champions. Quarterly results are transmitted to the NDNQI website where the data undergoes a systematic quality assurance process and review for outliers and inconsistencies between the data elements. Data are then summarized and published in a quarterly report that allows participating facilities to compare their results with results from previous quarters and with other similar characteristics hospitals across the nation [35]. On a yearly basis, UMH participates in the International Pressure Ulcer Prevalence Survey conducted in the month of February. PU data is recorded using the Hill-Rom Prevalence Survey Scranton form. The form is a review of patient demographics, medical information, and assessment of 24 body sites for identification of pressure ulcers. Scranton forms are sent to Hill-Rom for compilation of data and results are reported back to the facility with annual PU benchmark and individual PU facility prevalence rates.

### 2.3. UMH Demographics

Based on results of the 2014 Hill-Rom Pressure Ulcer Prevalence Survey: Total number of Patients Surveyed (n = 305), Total number of Patients with Pressure Ulcers (n = 31), and Total number of Patients with HAPU (n = 2):

The majority of the 305 patients who participated in the survey were female (51%) and older than 50 years of age (80.7%); the most common weight reported amongst the participating patients was between 151 and 200 lbs. (46.3%).The most frequent Length of Stay (LOS) was less than 3 days (37%). Of all the surveyed patients, 8% had fecal incontinence and 8% urinary incontinence. Twenty five percent of patients had an indwelling catheter. Of all surveyed patients, 21% were at risk for PU according to the Braden Scale and 13.1% were considered at risk based on other risk/clinical factors and all the patients at risk (n = 104, 95.2%) had preventive measures utilized. Eighty percent of patients at risk received nutritional support.

The majority of the 31 patients who presented with PUs during the survey were female (71%) and older than 50 years of age (91%), with the majority being between 80 and 90 years of age. The most common patient weight in the PU population was between 151 and 200 lbs. (42.9%). The most frequent LOS in this group was 8–11 days (22.6%). In the PU group, 29% had fecal incontinence and 15% of patients had urinary incontinence. Forty-four percent of patients had indwelling catheters. All patients in the PU group had 100% preventive measures instituted in the past 24 h before the survey took place. 90.3% of patients received nutritional support. A total of 53 pressure ulcers were identified during the survey, (Stage I = 1), (Stage II = 18), (Stage III = 11), (Stage IV = 4), (Unstageable = 13), (Deep Tissue Injury = 4), (Undeterminable = 2).

The two patients who developed HAPU were one female and one male. One patient fell between the 50–59 years of age category and the other patient was within the 70–79-age category. Both patients’ weights were between 151 and 200 lbs. The average LOS in the HAPU patients was between 16 and 19 days. In the HAPU group, 50% had fecal incontinence and 50% urinary incontinence. One patient had an indwelling catheter. The Braden score at Admission for one of the HAPU patients was recorded as High Risk (10–12) and for the other patient Low Risk (15–18). Only one of the HAPU patients had received nutritional support. Both HAPU patients were identified with 3 Stage II PUs.

### 2.4. Intervention

The initial challenge in HAPU prevalence was addressed on multiple fronts at UMH including the following:

(1) Replacement of all inpatient support surfaces with Hill-Rom Advanced Microclimate ^®^ (Hill-Rom, Chicago, IL, USA) Technology mattresses, considered the next generation of low air loss which is thought to remove excess heat and moisture helping patients skin remain dryer.
(2) Implementation of a new comprehensive plan for assessment and monitoring of patients with HAPUs, which included the photographing of all wounds on Wednesdays for ongoing evaluation and management.
(3) Support surfaces replacement in the operating room for prevention of deep tissue injury and skin breakdown.

In addition, with the input of the committee, several nursing staff-driven protocols were implemented. Examples of these protocols included:

(1) One protocol triggered nurses to implement preventive measures on patients who scored 18 or below on the Braden Scale, and to initiate treatment of either stage one or two pressure ulcers. The protocol defined the specific plan of care for those patients. The nursing staff was empowered to initiate wound care treatment without waiting on consultation from the wound care nurses.
(2) A second protocol established a hospital-wide monthly and quarterly pressure ulcer surveillance process. The surveillance included specific unit rounding logs. The logs specifically identified patients who were admitted with either a “present on admission” (POA) or who developed a “hospital acquired pressure ulcer” (HAPU). The wound care committee reviewed these data and each inpatient nursing unit was informed of their hospital acquired wound rate. This information is then placed on the unit specific hospital pillar boards quarterly for all staff to review. The committee encouraged the managers and directors to perform weekly morning huddles addressing their wound care issues. To broadly engage the staff with participation, those inpatient units who had achieved zero HAPU rates were rewarded with monthly lunches.
(3) The committee reinforced “repositioning” as a basic tenet of nursing care to prevent pressure ulcers. Most repositioning policies are based on historic recommendations and supported by current best practice guidelines [36].
(4) Mallah *et al.* also recommends repositioning the bed-ridden patient every two hours to help eliminate interface pressure ulcer development [15].
(5) In order to enable our staff to reposition the bed-ridden patient every two hours, the committee sought the support of executive directors and nurse managers to increase the number of nurse assistants on each unit to three or four and to enforce hourly rounding to bedridden patients. The newly hired staff was utilized to aid the non-bedridden patients get out of bed to chair—critical to the success of meeting the intent of a repositioning protocol.

**Figure 1:**
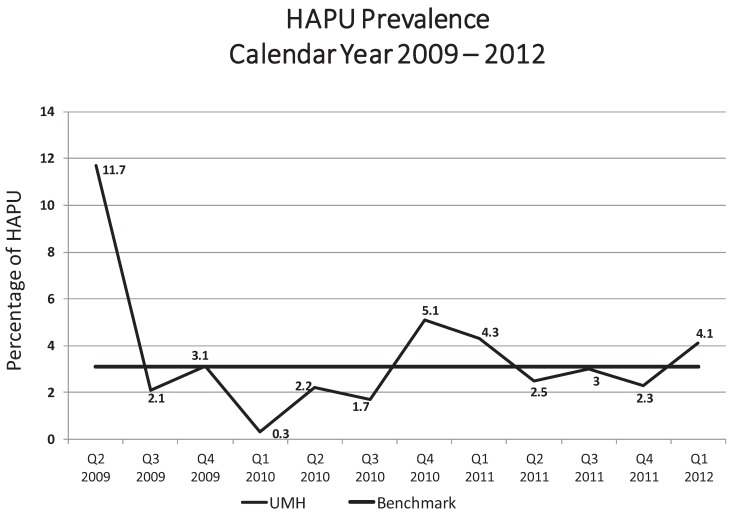
UMH Hospital-wide quarterly prevalence of HAPU [38].

These interventions proved to be effective in decreasing the prevalence of HAPU, as noted in Figure 1. The initial rate of HAPU prevalence of 11.7% in April 2009 was clearly concerning. Hill-Rom Survey benchmarks for HAPU in 2009 was reported at 3.1% for similar acute care settings [37].

Following the initial interventions described above the rate dropped to 2.1% and the prevalence of HAPU at UMH remained low until Q4 of 2010 when the rate rose to 5.1% which prompted staff re-education and wired multidisciplinary rounds. Consequently, HAPU rates decreased over the next four quarters but rose again to 4.1% by Q1 of 2012.

It became clear to the multidisciplinary team that extraordinary vigilance would be required to approach the theoretical goal of zero HAPU. In order to become more successful, the team had to go well beyond the basics. Remarkably, additional success has been achieved since 2012 by focusing upon the introduction of new skin care products as well as continual education.

Interestingly, when the team first analyzed issue commonalities between units they came to the conclusion that each unit was experiencing the same issues with the incumbent skin care products that impacted staffing. The encountered problem was that the zinc based protective barrier cream that was being used was very difficult to remove from the patient’s buttocks and sacral areas. This required the staff to apply different lotions to remove the zinc-based cream or to wash the body parts with soap and water, which resulted in skin irritation and excoriation. This resulted in additional staff time and was not cost effective as additional products such as lotions or ointments were used to remove the zinc-based product. The committee became focused upon skin care issues such as this and as a result:

(1) Skin care policies were implemented to standardize the prevention and care of all HAPU.
(2) Re-education on Braden Scale was mandated in order to identify patients at higher risk of HAPU and to initiate early intervention.
(3) A wound, ostomy, and continence (WOC) nurse was added to staff to lead and coordinate the skin maintenance and management programs and to provide continuous education and training for the patient care staff. The WOC nurse holds monthly product evaluations, education, and review with wound care champions representing the various hospital units.

The committee also realized the importance of trialing innovative products. Algorithms were developed for these new products to prevent and treat skin breakdown. As a team, products from various companies were researched and trialed. Our sister hospital, University of Miami’s Sylvester Comprehensive Cancer Center, had implemented a new skin care product line the prior year which had proven very successful. The product line involved the use of silicone emollient-based skincare products, augmented with components important for nourishment of skin (Remedy^®^, Medline Industries, Mundelein, IL, USA). While consulting at Sylvester, UMH wound care nurses identified improvement in wound care and pressure ulcer prevention with this product line. The wound care nurse feedback was reviewed and presented at the UMH wound care committee meeting. The committee’s decision was to evaluate these products hospital-wide.

Despite increasing research on the effectiveness of preventive measures developed in recent decades, there is still a knowledge deficit in PU prevention among health care personnel [34,35,36,37,38]) Recognizing this knowledge deficit the nursing staff was mandated to complete an online course that was provided by the skincare product provider as a foundational component of the PUPP program (Medline Industries, Mundelein, IL, USA) [39].

Education was available to all Registered Nurses (RNs) and Certified Nursing Assistants (CNAs) via a web-based suite of interactive educational materials. The program also included quick reference training tools in order to implement an operative pressure ulcer prevention program immediately and commence reducing the prevalence of pressure ulcers among the patients. These training materials were in the form of quick study guides, workbooks, and patient and family education brochures which were published in the hospital internal website for easy access by staff members. The course was administered through UMH’s organizational learning department and the online education included a pre-test and post-test of PU knowledge. In addition, the skincare product representatives were available daily to educate the UMH nursing staff on proper use of said products. The skincare product educators also attended daily nursing huddles to engage the staff with in-services and real-time demonstrations. The wound care committee representatives were ultimately responsible to reinforce ongoing teaching of the nursing staff.

## 3. Results

The education implementation was put in place with full commitment from hospital educators, wound care champions and specialists, clinical resource management staff, clinical pharmacists and hospital leaders. A total of 838 registered nurses and nursing assistants from the in-patient, emergency room, and procedural areas were enrolled to take pre-test and post-test evaluations of pressure ulcer knowledge. Compliance in completing the education and testing was monitored weekly by the hospital’s education department and reports were sent to the staff’s managers and directors. At the end of the two-month enrollment period 99% compliance rate was achieved. Average post-test results after program implementation increased by 14% among the nurses and 23% among the certified nursing assistants (see Table 1).

**Figure 2:**
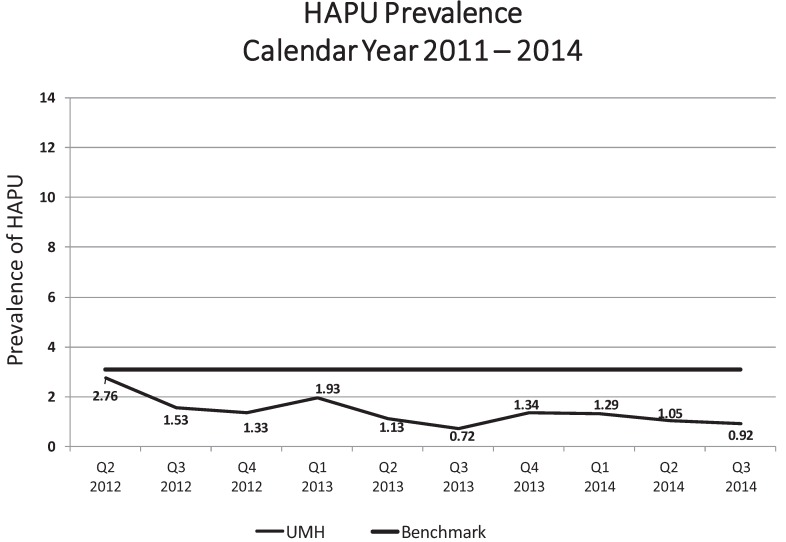
UMH Hospital-wide 2012–2015 prevalence of HAPU.

**Table 1:** UMH PUPP Registered Nurses and Certified Nursing Assistants Pre and Post Education Test Results.

Program Participants	Average Pre-test %	Average Post-test %
Registered Nurses	78%	92%
Certified Nursing Assistants	75%	98%

Furthermore, all new nursing staff are required to attend a mandatory orientation class which specifically reviews all of our wound care protocols, policies, and computer-based learning on wound care. On an annual basis all nursing staff must complete wound care computer-based learning.

Most impressively, however, the prevalence of HAPU dramatically decreased (see Figure 2). The prevalence of HAPU decreased from 4.1% in Q1 of 2012, in Figure 1, to 2.8% by September, 2012 and has remained at 2.8% or below for 10 consecutive quarters. HAPU rates were between 1% and 2% demonstrating the enormous benefit of the structured skin care program with new products plus education.

## 4. Discussion

### Challenges and Successes

Staff commitment and “buy in” to the overall program educational components was identified early on as a key challenge. Incentives such as reward packages to the unit who had the highest percentage of program completion on a weekly basis were offered. In addition, hospital educators scheduled day and night computer laboratory hours during meal breaks and low volume nursing times to assist staff to complete one or two modules at a time. Wound care champions provided RN staff coverage to allow the bedside nurse an opportunity to complete on line modules. Increased knowledge regarding HAPUs was equated to increased job satisfaction as one of the positive feedbacks received from program participants. After completion of the educational program, staff commented positively regarding new learning as well as the helpful review of information they had already forgotten. The participants received a certificate of completion and recognition pins after completing their modules. The result has been an increase in staff engagement in identification and management of HAPUs leading to improved staff satisfaction and outstanding patient outcomes.

Given the strong organizational foundation of the UMH of the PUPP program, with implementation of: nurse driven policies, education, new products, increased nursing staff, hourly rounding, emphasis on repositioning of bed ridden patients, and monitoring of pressure ulcer compliance, resulted in a dramatic improvement in the HAPU prevalence rate in our facility. Most importantly, additional success resulted from the subsequent introduction of new skin care products plus dedicated education and instruction surrounding the use of those products along with general PU education. The result is excellent patient outcomes at UMH with HAPU prevalence below benchmark for over 10 consecutive quarters and prevalence rates approaching zero HAPU for several of those months. The PUPP program from the skincare provider has evolved into a more comprehensive program (Skintegrity^TM^, Medline Industries). It would be of interest to probe the efficacy of this new comprehensive program in future research studies.

## 5. Conclusions

At UMH we were able to maintain HAPU rates well below its targeted benchmark of 3.1% for 25 consecutive months in part because of the utilization of new skin care products and continuing re-education. Our experience at UMH suggests, however, the key elements of a Pressure Ulcer Prevention Program (PUPP): (1) Implementation of Evidence-Based practices; (2) Evidence-Based product selection; and (3) Clinician education, only form the foundation for a successful PUPP. Improved outcomes can only be achieved with ongoing vigilance, oversight, strategic commitments, and institutional support—which means going well beyond the basics.
